# Associations Between Milk Composition, Blood Metabolomics, and Systemic Physiological Indices in High- vs. Low-Yielding Guanzhong Dairy Goats During Early Lactation

**DOI:** 10.3390/vetsci12100990

**Published:** 2025-10-14

**Authors:** Ziqi Meng, Chenxi Fang, Qinan Zhao, Lei Yang, Hai Jin, Jingwei Qi, Xiaoping An

**Affiliations:** 1 College of Animal Science, Inner Mongolia Agricultural University, Hohhot 010018, China; 18047131906@163.com (Z.M.); fangchenxi@emails.imau.edu.cn (C.F.); 2 Key Laboratory of Intelligent Animal Husbandry in Universities of Inner Mongolia Autonomous Region, Comprehensive Research Platform for Intelligent Animal Husbandry in Universities of Inner Mongolia, Research Center for Feed Engineering Technology of Herbivorous Animal Husbandry in Inner Mongolia, Hohhot 010018, China; 3 Inner Mongolia Academy of Agriculture and Animal Husbandry Sciences, Hohhot 010031, Chinacars39@163.com (L.Y.);

**Keywords:** dairy goats, early lactation, milk yield, untargeted metabolomics

## Abstract

**Simple Summary:**

This study quantified milk yield divergence in Guanzhong dairy goats managed under identical early-lactation conditions. High-yielding animals produced more milk despite a slightly lower lactose percentage, resulting in greater daily lactose output. Their serum contained less total protein and glucose but higher concentrations of specific antioxidants and immune markers. Metabolomic screening further identified blood metabolites tightly linked to efficient energy utilization and milk synthesis. These findings unveil a physiological strategy whereby HY goats prioritize nutrient allocation to the mammary gland while sustaining superior health, offering metabolic benchmarks for genetic selection and precision feeding that simultaneously increase production and animal welfare.

**Abstract:**

This study aimed to elucidate the intrinsic regulatory mechanisms by comparing milk quality, blood metabolomics, and physiological indices between high-yielding (BH, *n* = 15, high milk yield, daily milk yield with 4.08 ± 0.17 kg) and low-yielding (BL, *n* = 15, low milk yield, daily milk yield with 2.54 ± 0.26 kg) Guanzhong dairy goats during early lactation. The results showed that the lactose content in the BH group was significantly lower than that in the BL group (*p* < 0.05), but the total daily lactose yield was 60 g higher. No significant differences were observed in milk fat or milk protein (*p* > 0.05). Among blood biochemical indices, total protein (TP), glucose (GLU), and alkaline phosphatase (ALP) were significantly lower in the BH group (*p* < 0.05), while β-hydroxybutyrate (BHBA) was significantly higher (*p* < 0.05). Milk yield exhibited a highly significant negative correlation with TP and creatinine (CRE). Regarding immune and antioxidant indices, catalase (CAT), glutathione peroxidase (GSH-Px), IgM, and IL-2 were significantly elevated in the BH group (*p* < 0.05), while IL-6 was significantly reduced (*p* < 0.05). CAT and IL-2 showed positive correlations with milk yield. Using a subset of animals for in-depth profiling (*n* = 6 per group)Serum metabolomics identified 184 differential metabolites (114 upregulated, 70 downregulated). In the BH group, betaine, acylcarnitines, and L-valine exhibited significant negative correlations with milk yield, implicating pathways related to fatty acid -oxidation, methyl donor regulation, and amino acid metabolism. These findings indicate that high-yielding dairy goats achieve efficient lactation through enhanced fatty acid β-oxidation, optimized methyl donor regulation for milk fat synthesis, and prioritized allocation of amino acids towards the mammary gland.

## 1. Introduction

Feed efficiency is a core indicator for evaluating the production benefit of dairy animals and an important trait in breeding programs [1,2]. As the third largest milk source globally [3], goat milk not only is rich in high-quality protein, medium-chain fatty acids and various minerals, but also occupies an important position in the dairy market due to its characteristics such as small milk fat globules, moderate lactose content and human-like milk protein structure [4,5]. With the upgrading of consumption and the enhancement of health awareness, the demand for goat milk continues to grow [6]. However, feed cost accounts for about 60–70% of the total breeding cost in large-scale dairy goat farms and has shown an upward trend in recent years, making improving feed conversion efficiency a key goal in breeding and production management, which has been included in the comprehensive selection index by European and American countries [7,8,9]. Early lactation is a critical physiological stage for mammary gland functional recovery and the establishment of lactation potential in dairy goats. Nevertheless, significant differences in lactation performance persist among individuals under identical feeding conditions including the same feed and dry matter intake, and similar physiological states, such as parity, body condition, and health status [10]. This variation in milk yield beyond nutritional input regulation suggests the role of intrinsic physiological states and metabolic regulatory mechanisms. Previous studies have identified specific serum metabolites, such as certain acylcarnitines, amino acids, and methyl-donors like betaine, as potential biomarkers for lactation performance, as they are intricately involved in energy metabolism and milk precursor synthesis [11,12]. A close relationship is therefore postulated to exist between milk components and key serum physiological indicators and such specific metabolites. These differences and associations together constitute the physiological basis of lactation differentiation and reflect its regulatory mechanism. Understanding the physiological basis of this intrinsic variation is crucial for unlocking lactation potential, enhancing feed efficiency, and enabling precise breeding selection. We hypothesized that the divergence in lactation performance between high- and low-yielding goats is underpinned by distinct intrinsic metabolic regulation, which orchestrates optimized nutrient partitioning towards the mammary gland and creates unique systemic metabolite profiles. This study systematically compared differences in milk quality, blood biochemical indices, immune status, antioxidant capacity, and serum metabolites between high-yielding (BH) and low-yielding (BL) dairy goats. It further analyzed the associations between milk composition and key serum physiological indices/metabolites. Based on significant correlations between milk yield and critical blood parameters, integrated data analysis aimed to reveal biomarkers and intrinsic mechanisms underlying lactation performance differences, providing a basis for optimizing milk yield and health management throughout the lactation cycle.

## 2. Materials and Methods

### 2.1. Animals

The animal experiments involved in this study were approved by the Animal Ethics Committee of Inner Mongolia Agricultural University (Approval No. NND2023117) on 7 November 2023. This experiment was conducted at Shengjian Shenda dairy goat farm in Hohhot, Inner Mongolia Autonomous Region, China. From 216 lactating Guanzhong dairy goats in the same pen, 96 individuals with 2~3 lactations, producing twins, in similar body condition and without lactation abnormalities such as mastitis, single or multiple teats were initially screened. 41 goats were screened based on the stability of the lactation growth trend, the exclusion of fluctuating abnormal individuals and the similarity of feed intake (1.92 ± 0.28 kg) and body weight (52.21 ± 5.75 kg). To further ensure differences between groups, individuals with intermediate lactation were excluded and 30 clinically healthy goats were finally selected for inclusion in the formal trial. These 30 goats were divided into 2 experimental groups: based on the average daily milk yield calculated from the recordings at 10, 20, and 30 days postpartum, group 1 was a high yielding group (BH, *n* = 15) with an average lactation yield of 4.08 ± 0.17 kg/day and group 2 was a low yielding group (BL, *n* = 15) with an average lactation yield of 2.54 ± 0.26 kg/day.

All the test goats were kept in the same environmental enclosure and fed a total mixed ration (TMR) prepared according to NRC (2007) Nutritional Requirements of Small Ruminants (NRSRSR), the composition and nutrient content of the diets are shown in Table 1. The TMRs were prepared in a special mixing station, and the raw materials were weighed and put into the diets according to the formula, and then the diets were mixed thoroughly (about 10–15 min). and then fed to the test goats at fixed times (08:00 am, 16:00 pm) at about 2.5 kg DM/head/day, and adjusted according to the rate of leftovers the actual daily feed intake was monitored by weighing the feed offered and the refusals before the morning feeding to calculate daily dry matter intake to ensure ad libitum feeding, and the troughs were thoroughly cleaned before feeding every day. An automatic waterer was installed in the goat house to ensure 24-h free access to water, and was cleaned and disinfected on a regular basis. Goat beds are covered with dry and clean bedding, which is regularly cleaned and replaced to maintain hygiene. The goats were inspected daily by professional staff for their general health and behavior. No clinical signs of metabolic or inflammatory diseases were observed during the experiment. All goats were milked by an in-line milking machine according to standardized procedures, and the equipment was kept clean and regularly maintained.

### 2.2. Blood Sample Collection and Analysis

Before morning feeding and milking, 5 mL of venous blood was collected from the static artery using a disposable vacuum, centrifuged at 3000 r/min for 10 min, and the serum was divided and brought back to the refrigerator at −80 °C to be measured after liquid nitrogen quick-freezing. The following biochemical indexes were analyzed: total protein (TP), albumin (ALB), total cholesterol (CHOL), triglyceride (TG), low-density lipoprotein (LDL), high-density lipoprotein (HDL), glucose (GLU), urea (URE), creatinine (CRE), alkaline phosphatase (ALP), γ-glutamyltranspeptidase (GGT), methionine aminotransferase (AST), and other enzymes. transferase (AST) and alanine aminotransferase (ALT).

These indexes were measured by ELISA kits (Quanzhou Ruixin Biotechnology Co., Ltd., Quanzhou, China and Wuhan Genome Biotechnology Co., Ltd., Wuhan, China), and all the assays were performed in strict accordance with the instructions of the kits.

### 2.3. Milk Sample Collection and Analysis

The teats of the test goats were fully sterilized with iodophor and alcohol. Each day, milk samples were collected once in the morning and once in the afternoon into 50 mL centrifuge tubes, each containing one preservative tablet (Aiyuexing Tianjin Science and Technology Co., Ltd., TianJin, China). The morning and afternoon samples were then fully mixed at a ratio of 60%:40% and stored at 4 °C pending analysis. Prior to testing, the samples were restored to room temperature by incubation in a 38 °C water bath for 20 min. Subsequently, milk composition (including milk fat, milk protein, lactose, non-fat milk solids, and total solids) was determined using a Rapid Milk Composition Analyzer (LACTOSCANLWA; from Quanzhou Ruixin Biotech Co., Ltd., Quanzhou, China and Wuhan Genome Biotech Co., Wuhan, China) in strict accordance with the manufacturer’s manual. Each sample was measured three times, and the average value was recorded.

For statistical analysis, the milk composition data from each sampling day (at 10, 20, and 30 days postpartum) were analyzed. An average value for each milk component across the three sampling time points was then calculated for each individual goat. These individual averages (*n* = 15 per group) were used in all subsequent group comparisons and correlation analyses with blood parameters to avoid the issue of repeated measurements and to ensure statistical independence.

### 2.4. Statistical Analysis

Metabolites in serum and milk samples from high- and low-yielding dairy goats were characterized and quantified using ultra-performance liquid chromatography-tandem mass spectrometry (UPLC-MS/MS). The analysis was supported by the self-constructed database of Wuhan Meiterville Biotechnology Co., Ltd., Wuhan, China To identify metabolites with significant differences between the two groups, analysis of variance (ANOVA) was performed. Subsequently, all metabolic parameters were subjected to principal component analysis (PCA) to visualize the overall metabolic disparity. Prior to PCA, the data were standardized to unit variance, and principal components were extracted via singular value decomposition with missing value interpolation. The number of principal components retained for interpretation, along with their respective percentages of explained variance, is presented in the Section 3.

The effect of lactation volume on biochemical parameters of blood and milk samples was then explored using analysis of variance (ANOVA) combined with Duncan’s multiple comparisons in the generalized linear model (GLM) procedure in SAS 9.2 statistical software. All statistical models treated the animal as the experimental unit, with data representing the mean value for each individual (*n* = 30) to maintain independence of observations. Pearson’s correlation coefficient was used to determine the correlation between the milk sample and blood biochemical parameters.

For the untargeted metabolomics analysis, a randomly selected subset of serum samples (*n* = 6 per group, total *n* = 12) was used for high-throughput profiling. Metabolite data were combined with orthogonal partial least squares discriminant analysis (OPLS-DA) to screen for differential metabolites with VIP > 1 and pathway enrichment based on the KEGG database (hypergeometric test *p* < 0.05). Results are expressed as least squares mean ± standard error, with significance thresholds set at *p* < 0.05 (significant difference), 0.05 ≤ *p* < 0.10 (trend), and *p* ≥ 0.10 (non-significant differences). Quality control criteria: QC sample correlation |r| > 0.98, internal standard CV < 15%.

## 3. Results

### 3.1. Principal Component Analysis (PCA)

**Figure 1:**
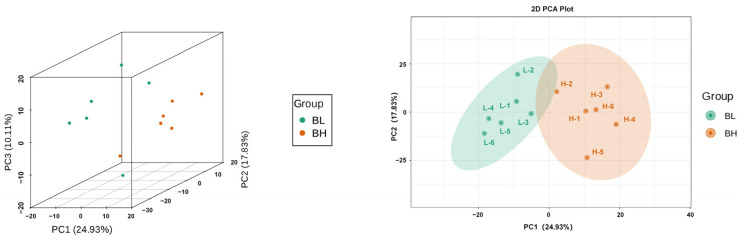
Principal Component Analysis.

The results of PCA are shown in Figure 1. There is a clear separation trend between the samples of BL and BH groups, and the samples of the two groups are different, which is in line with the logic of grouping to study the differences of metabolites.

### 3.2. Orthogonal Partial Least Squares Discriminant Analysis

**Figure 2:**
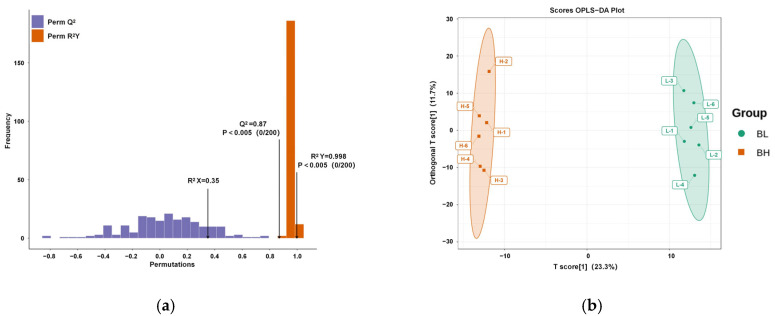
OPLS-DA model validation diagram. (**a**) Permutation test plot of the OPLS-DA model, showing (Q^2^ = 0.87), (R^2^Y = 0.998), and no overfitting. (**b**) OPLS-DA score plot, demonstrating clear separation of serum metabolomic samples from dairy goats with different body conditions (BH and BL groups), indicating significant differences in metabolites.

The OPLS-DA statistical method was applied to analyze the serum samples from the metabolomics subset (*n* = 6 per group) of dairy goats with different body conditions in early lactation. The OPLS-DA model was established as shown in Figure 2a, and the model parameters of BH and BL groups in this experiment: Q2 = 0.87, R2Y = 0.998. The predictive parameters indicated that the established OPLS-DA model had good predictive ability and stability excluding its overfitting. According to Figure 2b, the spacing between BH and BL groups was 23.3%, the gap within the group was 11.7%, the samples within the group were well aggregated, and the samples of both groups were clearly separated, which showed that there were significant differences in blood metabolites of dairy goats with different body conditions.

### 3.3. Metabolite Annotation

**Figure 3:**
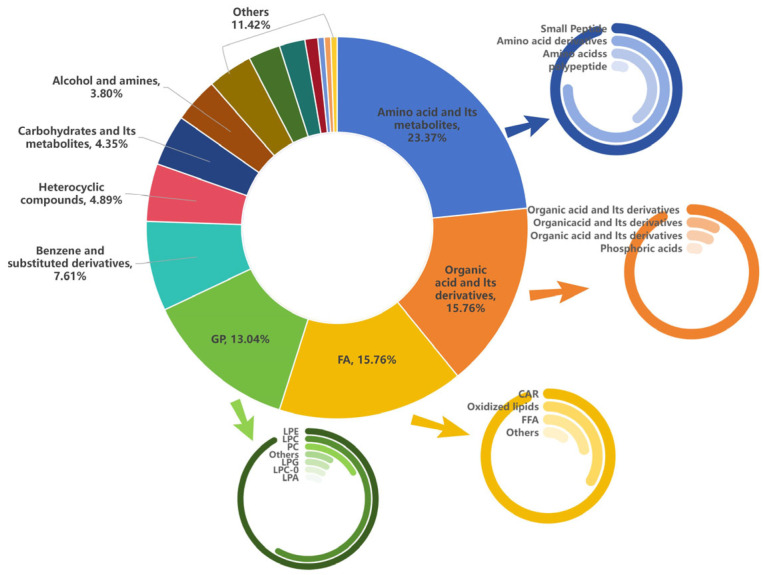
Composition of differential metabolites in BH and BL groups. Note: Each color represents a metabolite class, and the area of the color block indicates the size of the proportion of the class.

As shown in Figure 3, the processed metabolite mass spectrometry data were compared with the self-constructed database of Wuhan Meiterville Biotechnology Co., Ltd.Wuhan, China and public databases, resulting in a total of 746 metabolites detected in the serum of dairy goats with different lactation levels at the early stage of lactation, and the metabolites can be subdivided into 15 categories, mainly including: amino acids and their metabolites 23.37%, organic acids and their derivatives 15.76%, fatty acyls 15.76%, and glycerophospholipids 13.04%.

### 3.4. Screening of Differential Metabolites

**Figure 4:**
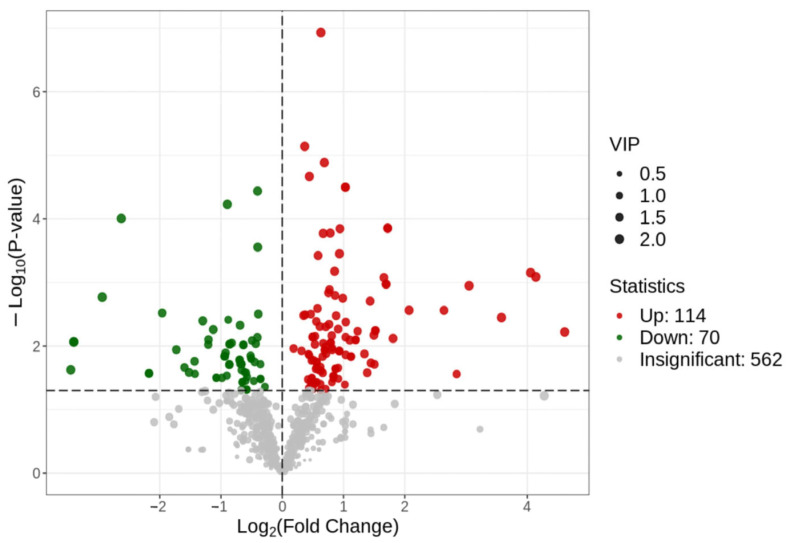
Volcanic map of BL and BH differential substances. Note: Scattered dots in the figure indicate the final screening results: red indicates that the metabolites are significantly up-regulated; green indicates that the metabolites are significantly down-regulated; and gray indicates that the metabolites are non-significant differences.

Differential metabolites of BL and BH groups are shown in Figure 4, a total of 184 differential metabolites were screened, of which 114 were up-regulated and 70 were down-regulated. The highest number of up-regulated metabolites were amino acids and their metabolites (39), followed by fatty acyls (20) and glycerophospholipids (10). The down-regulated metabolites were mainly organic acids and their derivatives (21), followed by glycerophospholipids (14) and benzene and its derivatives (9).

### 3.5. Differential Analysis of Milk Components of Dairy Goats at Different Lactation Levels

As shown in Table 2, the lactation amount of the BH group was significantly higher than that of the BL group (*p* < 0.0001), the lactose content of the BH group was significantly lower than that of the BL group (*p* < 0.05), and other milk composition indexes did not produce significant differences between different groups (*p* > 0.1).

### 3.6. Analysis of Differences in Early Lactation Blood Biochemical Indices of Dairy Goats and Their Correlation with Dairy Quality

From Table 3, it can be seen that among the blood biochemical indices, the levels of TP, GLU, ALP, and GGT in the BH group were significantly lower than those in the BL group (*p* < 0.05). The level of BHBA in the BH group was significantly higher than that in the BL group (*p* < 0.05); the level of CRE in the BH group showed a tendency to be lower than that in the BL group (*p* = 0.0799); and the level of FFA in the BH group showed a tendency to be higher than that in the BL group (*p* = 0.0809).

### 3.7. Analysis of Blood Antioxidant and Immune Levels in Dairy Goats with Different Lactation Levels

From Table 4, it can be seen that among the blood antioxidant levels, the levels of T-AOC, CAT, and GSH-Px in the BH group were significantly higher than those in the BL group (*p* < 0.05). In the blood immune levels, the levels of IgM and IL-2 in the BH group were significantly higher than those in the BL group (*p* < 0.05); the level of IL-6 in the BH group was significantly lower than that in the BL group (*p* < 0.05); and the level of IgG in the BH group showed a tendency to be higher than that in the BL group (*p* = 0.0799).

### 3.8. Correlation Between Blood Biochemical Indexes and Milk Quality of Dairy Goats at the Early Stage of Lactation

**Figure 5:**
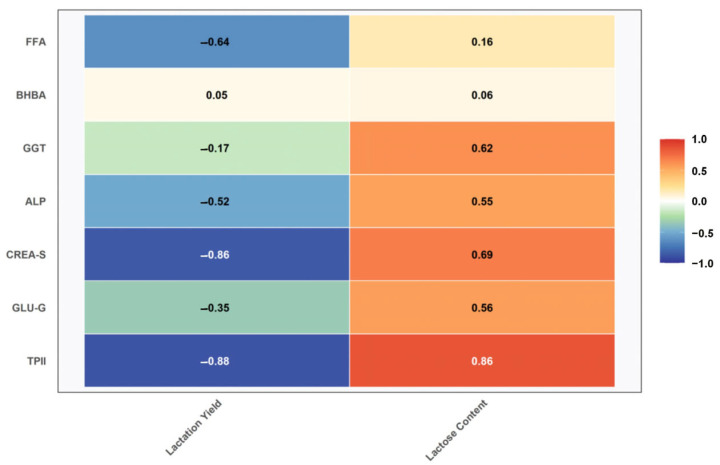
Correlation between blood biochemical indexes and milk quality of dairy goats in the early lactation period.

The correlation between blood biochemical indexes and milk quality of dairy goats in the early stage of lactation is shown in Figure 5, which shows that the lactation amount of dairy goats was significantly negatively correlated with total blood protein (r = −0.880, *n* = 30), significantly negatively correlated with creatinine (r = −0.861, *n* = 30), and the total blood protein content was significantly positively correlated with lactose (r = 0.861, *n* = 30).

### 3.9. Correlation Between Blood Immuno-Antioxidant Indexes and Milk Quality of Dairy Goats in the Early Lactation Stage

**Figure 6:**
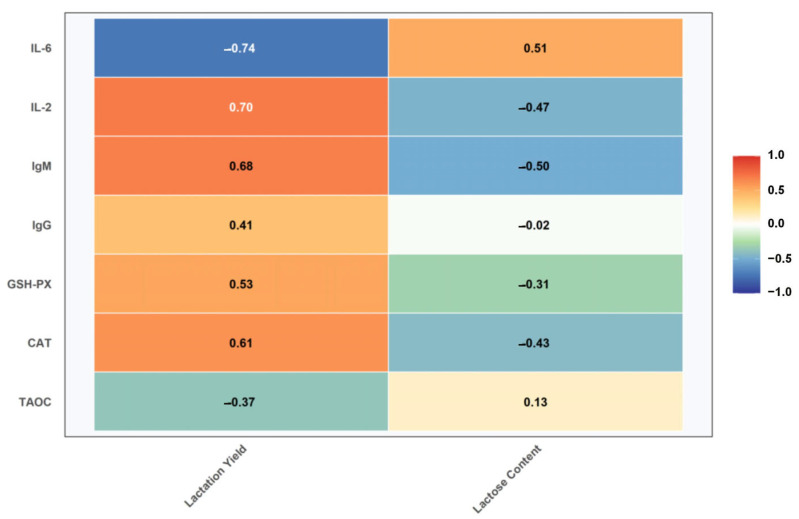
Correlation between blood immuno-antioxidant indexes and milk quality of dairy goats in the early stage of lactation.

The correlation between blood immuno-antioxidant indexes and milk quality of dairy goats in the early stage of lactation is shown in Figure 6, and the results showed that the indexes that showed significant correlation with lactation amount of dairy goats included CAT (r = 0.612, *n* = 30), GSH-PX (r = 0.533, *n* = 30), IgG (r = 0.407, *n* = 30), IgM (r = 0.684, *n* = 30), IL-2 (r = 0.701, *n* = 30), IL-6 (r = −0.738, *n* = 30), and indicators that showed significant correlation with lactose contained CAT (r = −0.427, *n* = 30), IgM (r = −0.502, *n* = 30), IL-2 (r = −0.467, *n* = 30), and IL-6 (r = 0.512, *n* = 30).

### 3.10. Correlation Between Key Serum Metabolites and Milk Quality of Dairy Goats in the Early Lactation Period

**Figure 7:**
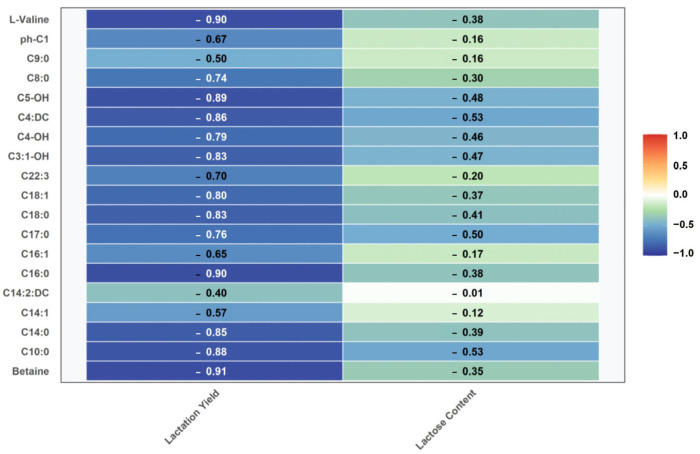
Correlation of key serum metabolites with milk quality in dairy goats in early lactation.

The correlation analysis between the key serum metabolites identified from the metabolomics subset and lactation performance of dairy goats in early lactation (within the same subset, *n* = 12) is shown in Figure 7, and the results showed that the top five metabolites most significantly correlated with lactation quantity were betaine (r = −0.909, *n* = 12), carnitine C16:0 (r = −0.900, *n* = 12), L-valine (r = −0.899, *n* = 12), carnitine C5-OH (r = −0.892, *n* = 12), and carnitine C10:0 (r = −0.875, *n* = 12). metabolites significantly associated with lactose included carnitine C10:0 (r = −0.526, *n* = 12) and carnitine C4:DC (r = −0.526, *n* = 12).

## 4. Discussion

### 4.1. Analysis of Milk Yield and Composition Changes in Dairy Goats During Early Lactation

The rate of milk yield increase during early lactation and the ability to sustain peak yield are core determinants of overall lactation performance in ruminants [13]. Typically, milk yield in dairy goats rises rapidly around 20 days postpartum, reaching peak lactation between 40–70 days [14,15]. Studies have found that the slope of the milk yield increase curve during early lactation can predict total lactation yield and is positively correlated with it [16]. In dairy cows, individuals with a rapid initial yield increase and longer peak persistence have significantly higher total milk production [17]. Furthermore, the full lactation curve of high-yielding dairy goats often exhibits a more pronounced ascending slope during early lactation [16]. This study collected data from a goat herd in early lactation. Does were categorized into high-yield (BH: initial high yield and fast growth rate, average increase >0.8 kg per 10 days) and low-yield (BL: average increase ≤0.5 kg per 10 days) groups based on milk yields at 10, 20, and 30 days postpartum. This dynamic assessment aids in judging full lactation potential, enabling early identification of high-yielding individuals and optimization of management strategies.

Milk yield and milk quality are key indicators of lactation performance. Milk protein, fat, and lactose are core nutritional components [18], their synthesis coordinated by spatial division of labor and multi-level regulation within mammary epithelial cells [19,20]. In this study, milk yield was significantly higher in the BH group, while lactose content was significantly lower compared to the BL group. This may be attributed to compensatory water secretion. However, when converted to daily lactose yield, the BH group produced approximately 173 g/d, about 60 g/d higher than the BL group. The energy cost per unit lactose produced might reflect optimized metabolic efficiency. Flores-Najera et al. [21] reported only a 0.1% difference in lactose percentage between high- and low-yielding crossbred dairy goats, but total daily lactose yield was significantly higher in the high-yield group. They suggested potential influences of nutrient partitioning priority and lactose dilution effects. Boshoff et al. [22] studied milk yield and composition changes across parities, finding that fourth-parity goats had significantly higher milk yield and total lactose yield compared to first-parity goats, confirming the positive correlation between total lactose yield and milk yield, consistent with our findings. González-Cabrera et al. [23] proposed that a lower lactose percentage is a physiological marker of high yield, possibly due to high intramammary pressure in high-yielding goats inhibiting lactose synthesis. In this study, milk protein and fat percentages did not differ significantly between yield groups. Scholtens et al. [24] found a high synergy between milk yield and fat yield in goats, with minimal fluctuations in fat percentage in high-yielding individuals, suggesting homeostatic regulation of mammary synthesis. This may be achieved by mammary glands maintaining fat synthesis homeostasis through acetate metabolism, making fat percentage less sensitive to yield fluctuations.

### 4.2. Analysis of Differences in Blood Biochemical Indices and Their Correlation with Milk Quality During Early Lactation

Blood serves as the primary source of nutrients for mammary synthesis. Its physiological and biochemical indices reflect animal health status and energy metabolism levels, directly influencing milk component synthesis [25,26]. Regarding energy metabolism, blood glucose (GLU) is a key indicator. During early lactation, substantial GLU is utilized for lactose synthesis to meet lactational demands. Studies indicate that the mammary gland can utilize up to 85% of plasma glucose for lactose synthesis [27]. The significantly elevated GLU in the BL group in this study may indicate inefficient lactose synthesis or may indicate reduced metabolic efficiency in glucose utilization. BHBA is a ketone body produced by incomplete oxidation of FFA in the liver. Its elevated concentration reflects the body’s compensatory response to an energy deficit via ketogenesis [28], while controlled FFA levels indicate precise regulation of fat mobilization to avoid metabolic disorders [29]. In this study, BHBA was significantly elevated in the BH group, while FFA showed a tendency to decrease, but both remained within normal physiological ranges (BHBA < 0.8 mmol/L). This suggests a strategy in high-yielding goats to compensate for glucose diversion to the mammary gland: selective activation of ketone body metabolism and precise regulation of FFA mobilization intensity, keeping hepatic BHBA production within physiological thresholds [27]. Regarding protein metabolism, TP is a key indicator of nitrogen resource allocation. The significantly lower TP in the BH group, coupled with its highly significant negative correlation with milk yield, and the reduced activity of GGT (a key enzyme for TP synthesis), reflect an optimization of protein partitioning via enhanced mammary uptake and suppressed hepatic synthesis. This aligns with the metabolic partitioning theory proposed by White et al. [30], suggesting that when cows face negative energy balance, the surge in mammary amino acid demand leads the liver to prioritize limited resources for energy homeostasis maintenance. Concerning liver function indicators, the decreased activity of ALP (a key enzyme regulating lipid transport), coupled with trends of lower LDL and higher HDL in the BH group, reflects a mechanism for directional lipid delivery to the mammary gland [31]. GGT, a core component of glutathione cycling, showed reduced activity, possibly indicating a shift in antioxidant capacity towards high-metabolism tissues like the mammary gland in high-yielding groups [32]. The significant reduction in ALP and GGT (*p* < 0.01) in the BH group reflects metabolic adaptation of liver function, while the lack of difference in AST and ALT rules out liver damage. Thus, high-yielding dairy goats optimize blood resource allocation through glucose diversion-ketogenesis compensation, mammary-prioritized protein uptake, and hepatic nutrient repartitioning to support efficient mammary lactation.

### 4.3. Analysis of Differences in Blood Immune and Antioxidant Indices and Their Correlation with Milk Quality in Dairy Goats with Different Lactation Levels During Early Lactation

The dynamic balance between oxidation and antioxidant systems is crucial for animal health. Imbalance leads to free radical accumulation, damaging immune function and biomembrane systems [33]. CAT and GSH-Px constitute the first line of defense against oxidative stress in mammals. CAT decomposes hydrogen peroxide (H_2_O_2_) preventing hydroxyl radical formation, while GSH-Px utilizes glutathione to reduce lipid peroxides, jointly maintaining cell membrane integrity [34]. In this study, both CAT and GSH-Px were significantly higher in the BH group and positively correlated with milk yield. Konvičná et al. [35] found that lactogenesis initiation in dairy cows, due to a dramatic increase in energy metabolism, causes mitochondrial overload and significantly elevates reactive oxygen species (ROS) levels. The body adaptively activates antioxidant systems to counter oxidative stress, with SOD activity rising continuously and GSH-Px rebounding significantly in mid-to-late lactation. As lactation level increased during early lactation, SOD and GSH-Px showed positive correlations, consistent with the positive regulatory trend of milk yield on antioxidant enzymes observed here.

Precise regulation of the immune system is vital for the health of lactating animals. Imbalance between pro- and anti-inflammatory responses can lead to immunopathological damage, manifesting as excessive inflammation or immunosuppression, impairing mammary mucosal barrier function and reducing milk component synthesis efficiency. IgM participates in mucosal immune defense, clearing pathogens early during invasion through mechanisms like complement cascade activation [36,37,38]. The highly significant positive correlation between IgM and milk yield in this study may reflect enhanced local mammary immunity in high-yielding individuals, further boosting systemic immunity. Silvestre et al. [39] found that higher metabolic activity in the mammary glands of high-yielding individuals might be accompanied by local immune activation, releasing more immune factors into systemic circulation. Consistent with our results, the significantly elevated IgM in the BH group, positively correlated with milk yield, may represent a compensatory upregulation of systemic immunity to cope with the physiological stress of lactational metabolism, safeguarding overall health and enhancing immune surveillance against mammary pathogens. IL-2 is a crucial immune factor promoting immune cell proliferation and differentiation. In dairy cows, IL-2 enhances local immune responses, maintaining mammary tissue health and indirectly promoting milk secretion. Adili et al. [40] found that supplementing heat-stressed lactating cows with autolyzed yeast additives significantly upregulated IL-2 gene expression and increased milk yield, aligning with the significant positive correlation between milk yield and blood IL-2 in our BH group. Elevated blood IL-2 might enhance mammary cell nutrient uptake, promote IGF-1 release, optimize lactose synthesis and immune energy utilization, and regulate immune responses to balance immune and lactational demands; it is plausible that such a mechanism could be operative, though further validation is needed, particularly given the sample size (*n* = 15 per group). Elevated IL-6 can prioritize energy consumption for the immune system, reducing resources available for lactation. Sustained high IL-6 levels might suppress appetite, impair liver function, and interfere with mammary function, thereby reducing lactation capacity. Trevisi et al. [41] studied the relationship between serum inflammatory cytokines and performance in Holstein cows, finding significantly higher serum IL-6 levels in high-inflammation groups, accompanied by significantly lower dry matter intake and milk yield, reflecting a significant negative correlation between elevated IL-6 and milk yield, consistent with our findings. In this study, blood IL-2 was significantly elevated in the BH group and positively correlated with milk yield, while IL-6 was significantly reduced and negatively correlated. These changes suggest that high-yielding dairy goats maximize lactation performance while safeguarding mammary health by strengthening local mammary immune responses and precisely regulating inflammatory balance.

### 4.4. Analysis of Differences in Serum Metabolome and Its Correlation with Milk Quality in Dairy Goats with Different Lactation Levels During Early Lactation

Serum metabolomics analysis identified 184 differential metabolites between high- and low-yielding dairy goats. These metabolites primarily implicated pathways related to choline metabolism, fatty acid oxidation, amino acid partitioning, and phospholipid remodeling, collectively regulating energy supply for mammary anabolism and precursor provision for milk components. Betaine, as a key methyl donor, activates the choline/betaine-homocysteine cycle, enhancing S-adenosylmethionine (SAM)-mediated methylation efficiency. This promotes the conversion of phosphatidylethanolamine to phosphatidylcholine, supporting milk fat globule membrane synthesis [42]. Haxhiaj et al. [43] found that elevated betaine levels might be a characteristic metabolic phenotype of low-yielding cows, while its downregulation in high yield might support milk production maintenance by optimizing energy allocation and immune balance. Wang et al. [11] reported that supplementing rumen-protected betaine in Holstein cow diets significantly increased milk yield, suggesting betaine can support lactation performance by sparing methionine. The downregulation of serum betaine in the BH group in this study may result from enhanced uptake by mammary tissue via high expression of betaine transporters. Acylcarnitine metabolism regulates energy supply through mitochondrial β-oxidation [44]. Yang et al. [12] and Wu et al. [45] found high-yielding cows reduced acylcarnitine levels to promote efficient fatty acid oxidation for lactational energy demands while reducing metabolic intermediate accumulation. Xu et al. [46] Our study provided reverse validation using an inflammation model, showing a negative correlation between acylcarnitine levels and lactation status. The downregulation of 17 acylcarnitine metabolites in the BH group aligns with this finding and may be a marker of optimized energy metabolism in high-yielding animals. Therefore, these acylcarnitines represent promising candidates for future validation as metabolic biomarkers for high lactation performance. High-yielding animals often require more valine to be directed towards mammary tissue for high-intensity metabolic consumption during milk fat and protein synthesis. Li et al. [47] found significantly lower L-Valine levels in rumen fluid of high-yielding cows compared to low-yielders. This aligns with the downregulation of blood L-Valine in the BH group goats observed here. High-yielding animals direct more nutrients like valine to the mammary gland for milk component synthesis, leading to reduced concentrations in some basal metabolic pools. Furthermore, Wang et al. [48] and Che et al. [49] support the critical role of valine in milk component synthesis, providing evidence for high-yield metabolic characteristics. This metabolic remodeling follows the principle of nutrient partitioning priority [50]. High-yielding dairy goats exhibit a metabolic signature characterized by the directed consumption of serum betaine, L-valine, and acylcarnitines, optimizing precursor supply for mammary synthesis through reprioritization of nutrient allocation.

## 5. Conclusions

This study demonstrates that during early lactation, high-yielding dairy goats redirect glucose to the mammary gland for lactose synthesis while activating ketogenesis to compensate for the systemic energy deficit. Concurrently, hepatic protein synthesis is suppressed to prioritize amino acid allocation for milk production. These metabolic adaptations are supported by an enhanced endogenous defense system, characterized by elevated antioxidant enzymes including catalase and glutathione peroxidase, and an optimized immune profile marked by increased IL-2 and IgM alongside decreased IL-6. Serum metabolomics identified a distinct metabolic phenotype in high-yielding goats, defined by significantly lower levels of betaine, specific acylcarnitines such as carnitine C16:0 and C10:0, and L-valine, which collectively facilitate methyl donor supply, efficient fatty acid beta-oxidation, and amino acid provision for milk synthesis. The consistent negative correlations of these metabolites with milk yield underscore their role in reprioritizing nutrient partitioning toward the mammary gland. We propose that the identified blood metabolite signatures and immuno-antioxidant features provide a concrete set of biomarkers for the early selection of elite dairy goats. Furthermore, these findings offer a physiological basis for developing precision feeding strategies that directly support the metabolic and immunological demands of high-yielding animals, thereby enhancing both productivity and herd health.

## Figures and Tables

**Table 1:** Composition and nutrient levels of diets (DM basis).

Items	Ingredients
Ingredients, %	
Peanut vine	7
Corn stalk	15
Sunflower seed skin	10
Alfalfa meal	8
Corn grain	37
Soybean meal	8
Germ meal	3.5
Cotton meal	1
DDGS ^1^	4
NaCl	0.6
NaHCO_3_	0.3
Limestone	1
CaHPO_4_	0.5
NH_4_Cl	0.1
4%Premix ^2^	4
Total	100
Nutrients	
DE ^3^, MJ/kg	10.5
DM, %	86.5
CP, %	12
NDF, %	32.68
ADF, %	20.18
Ca, %	0.74
P, %	0.33

Note: DDGS ^1^, Distillers Dried Grains with Solubles. The premix ^2^ provided the following per kg of diet: VA 3500 IU, VD 200 IU, VE 20 IU; Fe 60 mg, Cu 2 mg, Zn 60 mg, Mn 45 mg, I 0.6 mg, Se 0.2 mg, Co 0.2 mg; NaCl 5 g. DE ^3^ was a calculated value, while other nutrient levels were measured values.

**Table 2:** Analysis of differences in milk composition of dairy goats with different lactation levels.

Items	Groups		SEM	*p*
	BL	BH		
Lactation Yield/kg	2.54 b	4.08 a	0.1489	0.0001
Milk Fat %	4.8	4.3	0.2063	0.2962
Milk Protein %	3.43	3.29	0.0747	0.5122
Lactose %	4.44 a	4.26 b	0.0407	0.0228
Non-fat milk solids %	8.67	8.46	0.1162	0.392
Solids %	13.54	12.86	0.3162	0.3305

Note: a, b different shoulder labels of peers indicate significant differences.

**Table 3:** Analysis of differences in blood biochemical indexes of dairy goats at different lactation levels.

Items	Groups		SEM	*p*
	BL	BH		
Total Protein (TPII)	85.68 a	78.12 b	1.6697	0.0189
Albumin (ALB)	22.87	22.45	0.4051	0.7407
Total Cholesterol (CHO)	2.24	2.39	0.0747	0.1945
Triglyceride (TG)	0.31	0.33	0.0145	0.4477
Low Density Lipoprotein (LDL)	0.69	0.64	0.0300	0.6382
High Density Lipoprotein (HDL)	1.24	1.4	0.0525	0.1093
Glucose (GLU)	2.72 a	2.33b	0.0707	0.0055
Urea (URE)	9.06	9.29	0.3266	0.6859
Creatinine (CRE)	29.36 x	26.33 y	0.8305	0.0799
Alkaline Phosphatase (ALP)	87.66 a	62.72 b	4.5899	0.0025
Gamma-Glutamyl Transferase (GGT)	63.01 a	51.97 b	1.9014	0.0019
Aspartate Aminotransferase (AST)	84.34	88.79	3.3935	0.6961
Alanine Aminotransferase (ALT)	5.45	5.04	0.3520	0.6813
β-HydroxyButyric Acid (BHBA)	358.76 b	445.01 a	22.7755	0.0082
Free Fatty Acid (FFA)	809.46 x	743.22 y	17.5576	0.0809

Note: The x, y means with in rows with different superscripts tend to be different (0.05 < *p* < 0.10). a, b means within a row with different superscripts tend to differ (0.05 < *p* < 0.10).

**Table 4:** Analysis of differences in blood antioxidant indexes of dairy goats at different lactation levels.

Item	Group		SEM	*p*
	BL Group	BH Group		
Antioxidant				
Total antioxidant capacity T-AOC (U/mL)	4.65 b	5.87 a	0.2435	0.0088
Catalase CAT (pg/mL)	312.31 b	405.39 a	17.2619	0.0042
Superoxide dismutase SOD (pg/mL)	617.37	627.29	29.9561	0.8726
Glutathione peroxidase GSH-Px (ng/mL)	3.03 b	4.31 a	0.2231	0.0011
Malondialdehyde MDA (ng/mL)	58.51	54.49	2.4924	0.4327
Immunization				
IgA (ng/mL)	4.43	4.92	0.1504	0.1072
IgG (μg/mL)	9.76 y	11.18 x	0.5330	0.0821
IgM (ng/mL)	7.31 b	8.96 a	0.4257	0.0334
IL-2 (Pg/mL)	69.07 b	88.44 a	4.0040	0.0144
IL-6 (Pg/mL)	107.49 a	93.67 b	3.3554	0.0379
IL-10 (Pg/mL)	79.57	73.39	2.8014	0.3737
TNF-α (pg/mL)	45.9	48.4	2.3664	0.6102

Note: The meanings of a, b, x and y are the same as those in Table 3.

## Data Availability

The original contributions presented in this study are included in the article material. Further inquiries can be directed to the corresponding author.
